# Disclosing Main authors and Organisations collaborations in bioprinting through network maps analysis

**DOI:** 10.1186/s13326-020-0219-z

**Published:** 2020-05-01

**Authors:** Leonardo Azael García-García, Marisela Rodríguez-Salvador

**Affiliations:** 1 University of Sussex, School of Engineering and Informatics, Falmer, Brighton, UK; 2 Tecnologico de Monterrey, Escuela de Ingeniería y Ciencias, Monterrey, Nuevo Leon Mexico

**Keywords:** Network map analysis, Betweenness centrality, Bioprinting, Text mining, Collaboration analysis, scientometrics, competitive technology intelligence

## Abstract

**Background:**

Scientific activity for 3D bioprinting has increased over the past years focusing mainly on fully functional biological constructs to overcome issues related to organ transplants. This research performs a scientometric analysis on bioprinting based on a competitive technology intelligence (CTI) cycle, which assesses scientific documents to establish the publication rate of science and technology in terms of institutions, patents or journals. Although analyses of publications can be observed in the literature, the identification of the most influential authors and affiliations has not been addressed. This study involves the analysis of authors and affiliations, and their interactions in a global framework. We use network collaboration maps and Betweenness Centrality (BC) to identify of the most prominent actors in bioprinting, enhancing the CTI analysis.

**Results:**

2088 documents were retrieved from Scopus database from 2007 to 2017, disclosing an exponential growth with an average publication increase of 17.5% per year. A threshold of five articles with ten or more cites was established for authors, while the same number of articles but cited five or more times was set for affiliations. The author with more publications was Atala A. (36 papers and a BC = 370.9), followed by Khademhosseini A. (30 documents and a BC = 2104.7), and Mironov (30 documents and BC = 2754.9). In addition, a small correlation was observed between the number of collaborations and the number of publications. Furthermore, 1760 institutions with a median of 10 publications were found, but only 20 within the established threshold. 30% of the 20 institutions had an external collaboration, and institutions located in and close to the life science cluster in Massachusetts showed a strong cooperation. The institution with more publications was the Harvard Medical School, 61 publications, followed by the Brigham and Women’s hospital, 46 papers, and the Massachusetts Institute of Technology with 37 documents.

**Conclusions:**

Network map analysis and BC allowed the identification of the most influential authors working on bioprinting and the collaboration between institutions was found limited. This analysis of authors and affiliations and their collaborations offer valuable information for the identification of potential associations for bioprinting researches and stakeholders.

## Background

Research articles are public documents that report scientific advancements to share knowledge and promote development in science. These documents contain fundamental information regarding not only to research but also to the organizations and authors involved. This data is of interest to identify leading organizations and to map scientific collaborations.

Scientometric tools such as co-citation analysis, bibliographic coupling, or co-author analysis can help to achieve these goals. Co-citation analysis and bibliographic coupling are mainly used to measure the flow of information based on the documents selected by authors, while co-author analysis is more focused on the analysis of collaboration between authors, taking into consideration the social aspect of the research collaboration. Furthermore, co-author analysis has been proved to be useful to determine the multi and interdisciplinary of the institutions and their collaborations [1]. Co-author analysis requires information related to authors’ aliases, affiliations, publications, areas of research, and their collaborations. This information can be obtained from digital libraries (DL) aimed to create systems for the identification of authors such as ORCID, which was created by non-profit organizations, or ResearcherID, Scopus, PubMED or Web of Science, which are companies that are developing their unique identifiers for authors [2–4]. When evaluating advances in science and technology, names of authors and affiliations become major indicators, as 1) their number of citations by peers correlates to their acknowledgment as influential on their area of research [5] and 2) contributes to determining the specific disciplines involved in the research [1], both are important elements to nurture the decision-making process. In this sense, Competitive Intelligence (CI) acquires a relevant role, through the definition, collection, analysis, and presentation of relevant information [6]. The CI process can be further enhanced by incorporating feedback form experts to validate the information obtained [7]. CI is fundamental to research and development (R&D), including products or processes with radical novelty, such as bioprinting.

Bioprinting is an emerging technology, a variant of additive manufacturing that involves the fabrication of 3D constructs for living tissues and organs [8, 9]. This discipline is growing at an accelerated pace, involving branches of knowledge such as biology and engineering. Bioprinting has been developed to assist the needs of a fast-growing population. This technique has potential social and economic impacts [10, 11], including a huge effect in organ transplants, where one of the main objectives is the printing of functional biological structures to help in the shortage of organs, thus overcoming long waiting lists and issues related to the transplanted organs such as rejection [10–12]. Although there have been significant signs of progress in the past years, there are some areas of research to be explored in this incipient technology [11]. Since academy and industry have acknowledged that bioprinting will have a significant impact on the health-care sector in the following years, the identification of technology trends in 3D bioprinting [13, 14], including potential printing techniques [15], becomes crucial to stay competitive and to develop new technologies in this field. With this aim, Rodriguez-Salvador et al. [7] performed a patentometric and scientometric analysis in bioprinting to identify trends and to explore the knowledge landscape of this technology. In addition, they also identified the most prolific institutions, being the MIT (113 publications) the number one, followed by Nanyang Technology University (103 publications), and Tsinghua University (93 publications); They also found that the three first countries with more publications were USA with 1491, followed by China with 744, and Germany with 377 [7]. These analyses are mostly based on the frequency of documents by affiliation and country, and no inclusion or exclusion terms were set. The insights obtained can be enhanced with the identification of the leading scientists and their field of expertise, thus distinguishing the principal areas of current research and determining potential opportunities for R&D.

In order to unveil scientific and technological trends, it is important to face big volumes of information using text mining. This activity can be applied to identify and extract potentially useful information from texts. It combines tools such as machine learning, artificial intelligence, and statistics to analyse large amounts of both structured and unstructured data. The information obtained can contribute to understanding patterns in data by making use of tools such as text categorization, text clustering, information extraction, among others [16]. Information retrieval, word frequency analysis, word distribution, pattern recognition, and visualisation techniques are some of the most frequent practices [17]. As a conclusion, text mining adds important value to the pattern recognition by structuring the content of data from textual sources for research, data analysis, business or competitive intelligence (CI) [17–19].

A fundamental topic for the CI is the determination of key players, such as the main organizations and authors involved in scientific advancements. Network analysis can be used to identify the collaboration in a visual pattern, where either the authors or affiliations are represented by nodes and their collaborations can be seen as the connection among them. Moreover, the nodes with common attributes of interest for the analysis can be grouped using clusterization. Clusterization allows to group components with similar characteristics, such as research topics or techniques. When clustering collaborations, the closer the nodes in authors or affiliations network maps, the more similarities they share [5, 20]. Furthermore, collaboration analysis can be strengthened with the assessment of the Betweenness Centrality (BC) to determine the level of association of the nodes according to their position in the network. A straightforward measurement of the association level can be the connectivity, but it fails to disclose the importance of a node. To overcome this, BC measure can be calculated to evaluate the importance of a node and its social interaction in a network as this measure counts the number of regions in the map connected by each element, setting their importance in the flow of information [21, 22].

Scientometric and patentometric techniques have been used recently to analyse the number publications per year, the main authors, and organizations to determine the main advancements in bioprinting (methods, materials, etc.) [23–25]. Scientometric and text mining can be used to detect the authors and affiliations with more publications and more influence in bioprinting. This information can be an input for people looking for well-known experts in bioprinting or state-of-the-art developments in the field.

To achieve the main goal of this paper, a customised search query was used to gather documents from Scopus. The query included keywords highly used in the most cited papers on bioprinting. Two network maps of collaborations, one for authors and one for affiliations, were generated and analysed. Further analyses were carried out to estimate the BC measurement, and the relationship between number of publications and the number of collaborations. These parameters were used for the identification of the most prolific (those with more publications on this topic) and important authors and institutions involved in the publications of advances in bioprinting.

This analysis is the first attempt to undertake a quantitative analysis using a network analysis approach and the calculation of centrality measurements to strengthen the CI methodologies. The findings enhance the perception of the importance of collaborations among institutions for the generation of high-quality scientific outcomes and for the dissemination of the knowledge generated, helping both researchers and stakeholders in the identification of potential opportunities for research and collaboration.

## Methods

This paper is focused on the network analysis of authors and institutions from scientific publications in bioprinting. The analysis comprises both, a network analysis on the collaboration among institutions and one that deals with the collaboration among authors. The network maps were generated in Gephi, an open-source software for network analysis [26–34]. Betweenness centrality was calculated for both, authors and institutions’ collaborations.

The adequate identification of specific keywords on the topic of interest is a determining step in the search strategy, as they contribute to the appropriate establishment of the search queries. A preliminary search in Scopus using only the term *bioprinting* with no period of time defined was the first stage of this research. Scopus was selected for the information retrieval as this is a major scientific database that includes information from more than 20,000 scientific journals [35]. The ten most cited papers identified through this search were selected, as they have been acknowledged as referents for the topic. Table 1, García-García[36], shows the ten articles that formed the first set of documents. These papers were used to identify the keywords to form the search queries. A text mining program was specially coded to carry out the text-mining of these publications, thus determining the most relevant keywords on the topic. With a broader range of terms and their synonyms we guarantee the inclusion of a wide range of publications compared to searches performed using only the term bioprinting. Three different types of keywords were searched in the whole text of the papers, being 1) the most frequent terms, 2) terms containing the word bio, and 3) the collocations, which are the juxtapositions of two words with a greater frequency. A cleaning of terms was accomplished manually afterward to sort them out according to specialized language of the subject. The identified keywords were separated by subtopics (i. e. technology, process, and application) to form the search queries. A set of 23 searches were performed with the selected terminology prior to the development of the definite query. These searches were used to identify the correct grouping of terms and the exclusion terms.

**Table 1:** Comparison of the top ten cited papers from Scopus obtained from the search of `bioprinting’ and the developed search query in titles, abstracts, or keywords

	Top ten results using the keyword *bioprinting* [36]					Top ten articles using the developed search string				
	Title	Authors	Year	Source	Cites	Title	Author	Year	Source	Cites
1	3D bioprinting of tissue and organs [38].	Murphy, S.V., Atala, A.	2014	*Nature Biotechnology* 32 (8), pp. 773–785	1498	3D bioprinting of tissues and organs [38].	Murphy S.V., Atala A.	2014	*Nature Biotechnology* 32 (8), pp. 773–785.	1498
2	Scaffold-free vascular tissue engineering using bioprinting [39].	Norotte, C., Marga, F.S., Niklason, L.E., Forgacs, G.	2009	*Biomaterials* 30 (30), pp. 5910–5917	600	Microscale technologies for tissue engineering and biology [40].	Khademhosseini A., Langer R., Borenstein J., Vacanti J.P.	2006	*Proc. Natl. Acad. Sci. U. S. A.*, 103 (8), pp. 2480–2487.	1163
3	3D bioprinting of vascularized, heterogeneous cell-laden tissue constructs [24].	Kolesky, D.B., Truby, R.L., Gladman, A.S., Homan, K.A., Lewis, J.A.	2014	*Advanced Materials* 26 (19), pp. 3124–3130	588	Clinical transplantation of a tissue-engineered airway [41].	Macchiarini P., Jungebluth P., Go T., Asnaghi M.A., Rees L.E., Cogan T.A., Dodson A., Martorell J., Bellini S., Parnigotto P.P., Dickinson S.C., Hollander A.P., Mantero S., Conconi M.T., Birchall M.A.	2008	*The Lancet* 372 (9655), pp. 2023–2030.	1014
4	Printing and prototyping of tissues and scaffolds [23].	Derby, B.	2012	*Science* 338 (6109), pp. 921–926	510	Mechanical properties and cell cultural response of polycaprolactone scaffolds designed and fabricated via fused deposition modelling [42].	Hutmacher D.W., Schantz T., Zein I., Ng K.W., Teoh S.H., Tan K.C.	2001	*Journal of Biomedical Materials Research* 55 (2), pp. 203–216.	939
5	Additive manufacturing of tissues and organs [43].	Melchels, F.P.W., Domingos, M.A.N., Klein, T.J., Bartolo, P.J., Hutmacher, D.W.	2012	*Progress in Polymer Science* 37 (8), pp. 1079–1104	495	Solid freeform fabrication of three-dimensional scaffolds for engineering replacement tissues and organs [44].	Leong K.F., Cheah C.M., Chua C.K.	2003	*Biomaterials* 24 (13), pp. 2363–2378.	739
6	25th anniversary article: Engineering hydrogels for biofabrication [45].	Malda, J., Visser, J., Melchels, F.P., Groll, J., Hutmacher, D.W.	2013	*Advanced Materials* 25 (36), pp. 5011–5028	465	Stem cell-based tissue engineering with silk biomaterials [46].	Wang Y., Kim H.-J., Vunjak-Novakovic G., Kaplan D.L.	2006	*Biomaterials* 27 (36), pp. 6064–6082.	657
7	A 3D bioprinting system to produce human-scale tissue constructs with structural integrity [47].	Kang, H.-W., Lee, S.J., Ko, I.K., Yoo, J.J., Atala, A.	2016	*Nature Biotechnology* 34 (3), pp. 312–319	466	Scaffold-free vascular tissue engineering using bioprinting [38].	Norotte C., Marga F.S., Niklason L.E., Forgacs G.	2009	*Biomaterials* 30 (30), pp. 5910–5917	600
8	Printing three-dimensional tissue analogues with decellularized extracellular matrix bioink [48].	Pati, F., Jang, J., Ha, D.-H., Kim, D.-H., Cho, D.-W.	2014	*Nature Communications* 53,935	412	Organ printing: Tissue spheroids as building blocks [49].	Mironov V., Visconti R.P., Kasyanov V., Forgacs G., Drake C.J., Markwald R.R.	2009	*Biomaterials* 30 (12), pp. 2164–2174.	594
9	Tissue engineering by self-assembly and bio-printing of living cells [50].	Jakab, K., Norotte, C., Marga, F., Vunjak-Novakovic, G., Forgacs, G.	2010	*Biofabrication* 2 (2),022001	290	3D bioprinting of vascularized, heterogeneous cell-laden tissue constructs [24].	Kolesky D.B., Truby R.L., Gladman A.S., Busbee T.A., Homan K.A., Lewis J.A.	2014	*Advanced Materials* 26 (19), pp. 3124–3130	588
10	3D Bioprinting of heterogeneous aortic valve conduits with alginate/gelatin hydrogel [51].	Duan, B., Hockaday, L.A., Kang, K.H., Butcher, J.T.	2013	*Journal of Biomedical Materials Research - Part A* 101 A(5), pp. 1255–1264	244	Binding and condensation of plasmid DNA onto functionalized carbon nanotubes: Toward the construction of nanotube-based gene delivery vectors [52].	Singh R., Pantarotto D., McCarthy D., Chaloin O., Hoebeke J., Partidos C.D., Briand J.-P., Prato M., Bianco A., Kostarelos K.	2005	*Journal of the American Chemical Society* 127 (12), pp. 4388–4396.	574

The search query was formed using the keywords previously identified in combination with Boolean and proximity operators, and exclusion terms. For this stage, the definite search was carried out by defining the period of time, from 1 January 2000 to 15 November 2017 (when the information collection was concluded). The main query is observed in the appendix A1. The collection activity involved the use of the query to search in title, abstract and keywords. A quick review of titles and abstracts of the documents found was carried out to discard those not related to bioprinting.

The bibliographic information of the documents identified in Scopus was retrieved and exported in a CSV format to be cleaned and analysed. A cleaning process and the complete normalization of the data was carried out to standardize authors and affiliations names. We performed a manual name disambiguation for both authors and affiliations. The two authors analysed manually all the names on each one of the publications gathered. Every time a similar name was observed, name disambiguation was carried out by looking to the full name, affiliation, and e-mail. The level of agreement on the disambiguation performed by the authors was measured using Cohen’s kappa [37]. Co-author analysis was limited exclusively to the information of the publications gathered and we did not require further information from available DLs.

The analysis to identify the most influential authors and affiliations was carried out by setting a threshold for each analysis. A threshold of five documents cited at least ten times was set for authors, while for the institutions we selected those with five documents cited at least five times. These inclusion parameters were based on the median number of cites for the whole set of documents, which was 10.33, and the median number of publications per author was 5.76. For institutions, the mean number of publications was 10 with the same median of cites for the documents, 10.33; however, only three affiliations were within the threshold, hence the median for citations and documents was reduced to 5 to include more affiliations.

To identify the most prolific authors, the top ten authors with more frequency within the threshold defined were selected and a Pearson correlation was computed to determine the relationship existing between the number of publications and the number of co-authors. The authors were clustered by the similarity of areas of research in the network maps and those with higher networking were identified by BC calculation. The number of times a node is taken as a connection for the shortest paths between two other nodes can be estimated through BC, which measures the node’s connection to different groups on a network map, being of a higher value the one who connects more groups [53]. The BC is obtained using the equation [53]:

$$ {C}_B(v)=\sum \limits_{v\ne s\ne t}\frac{\sigma_{st}(v)}{\sigma_{st}} $$

Where *σ*_*st*_ is the total of shortest paths from node *s* to node *t*, and *σ*_*st*_ (*v*) is the number of those paths that go through *v*.

The information within the threshold was imported into VOSviewer, a software for data analysis and visualisation [54, 55], to perform the network map analysis. The authors or institutions are represented by nodes or vertex in the network maps, and their connections are represented by links or edges; in this document, the terms are used indistinctly to refer to authors or affiliations and their connections. Two undirected network maps were constructed from two matrices, representing only the correlation and not causality. A matrix of authors and a matrix of affiliations were generated using the visualisation of singularities (VOS) of the VOSviewer software [55]. The clustering was performed in VOSviewer, computed using the default Field Independent Clustering Model (FICM) [55]. The statistical analysis to determine the BC of the nodes forming both maps was performed in Gephi. The final step of the analysis was the consultation with experts in 3D bioprinting to validate the results. Experts from UK and Asia were selected based on their international presence and impact in the field considering elements such as their number of cites, publications, projects, and their availability. Instead of providing the experts with a list of authors found on the results of this research, we asked them to provide a list of authors working on bioprinting according to their own criteria. This method was used to reduce bias in their selection, as they provided a list acknowledging their peers based on their own experience. Is it worth mentioning that the experts requested anonymity, therefore, we can only provide professional details of three of them at the time they were consulted. One of the experts was affiliated to the School of materials at the University of Manchester and had more than 10,000 Scopus citations. A second expert was affiliated to the Faculty of Engineering at the University of Nottingham and had more than 760 citations. A third one was affiliated to the Singapore Centre for 3D printing at Nanyang Technology University with more than 14,000 citations.

## Results

From the initial search, where the ten most cited articles in bioprinting from Scopus were considered, the top-cited article is *3D bioprinting of tissue and organs* [37]. This is a review of different techniques used in bioprinting cited 1498 times, as seen in Table 1; the second most cited article is *Scaffold-free vascular tissue engineering using bioprinting* [38]. This article describes a fully biological method to fabricate tubular vascular grafts and has been cited less than 50% of the first author, 600 times; the third paper, entitled *3D bioprinting of vascularized heterogeneous cell-laden tissue constructs* [24] was cited 446 times and describes methods to generate vascularized tissue constructs. The second and third papers are focused on one of the biggest challenges faced to print fully functional organs, the fabrication of scaffold-free blood vessels with mechanical properties close to the naturally grown vessels. Five of the ten articles were published in journals related to materials, four of them in general science journals (Nature Biotechnology, Nature Communications, and Science), and one in the journal of Biofabrication, as can be observed in Table 1. The results of the searches in Scopus using only the term *bioprinting* and those obtained using the search query developed are listed and compared in Table 1. It can be observed that the paper entitled *3D bioprinting of tissue and organs* still in first place in both results. The second paper listed in the results from the search string is *Microscale technologies for tissue engineering biology* by Khademhosseini et al. [39] with 77% of the cites of the most first publication, 1163, followed by the paper *Clinical transplantation of a tissue-engineered airway* by Macchiarini et al. [40], published in the Lancet. Interestingly, the first three papers are published in three major journals covering biology and medical-related science, and six out of the 10 papers on this search were published in journals related to materials and one in chemical engineering.

**Fig. 1:**
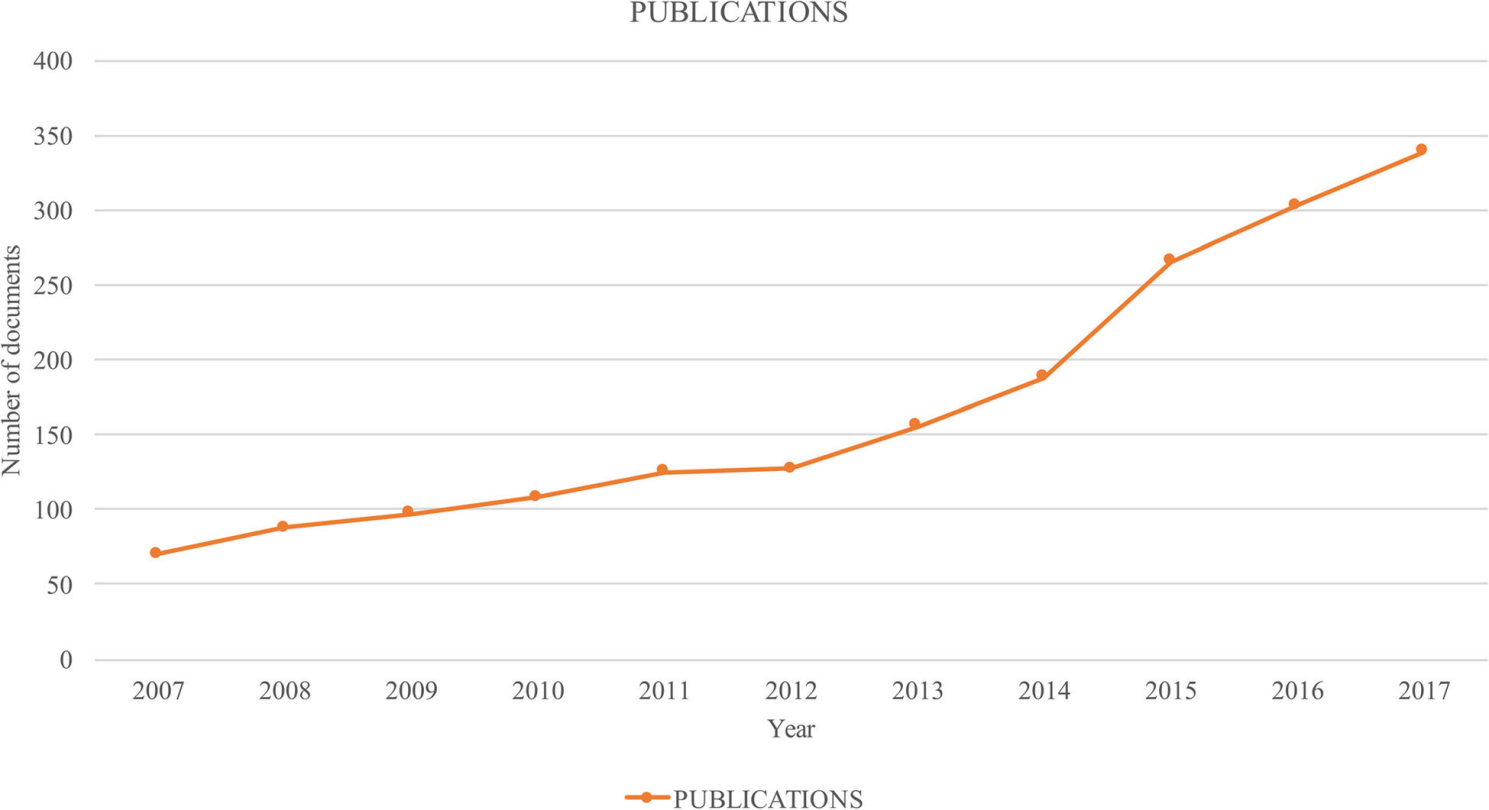
Publications per year in bioprinting

Using the previously defined search query a total of 2088 publications were found from 2007 to November 15 of 2017 (when information collection activity ended). Figure 1 shows the number of publications per year, there is a remarkable growth, where the highest number of publications is 339 for 2017.

After the data selection and cleaning, a total of 228 authors were found within the threshold of at least 5 documents with 10 or more citations. 89 of the authors were found with repeated surnames. A Cohen’s kappa (κ) of 0.62 was obtained for the agreement on the author name disambiguation. Values from 0.61 to 0.8 are ranked as Good [36]. A collaboration was observed in 93% of the authors, being 792 the total number of connections in the map. Regarding affiliations, a total of 20 organizations fall into the inclusion threshold, from which only 30% had an external collaboration.

**Fig. 2:**
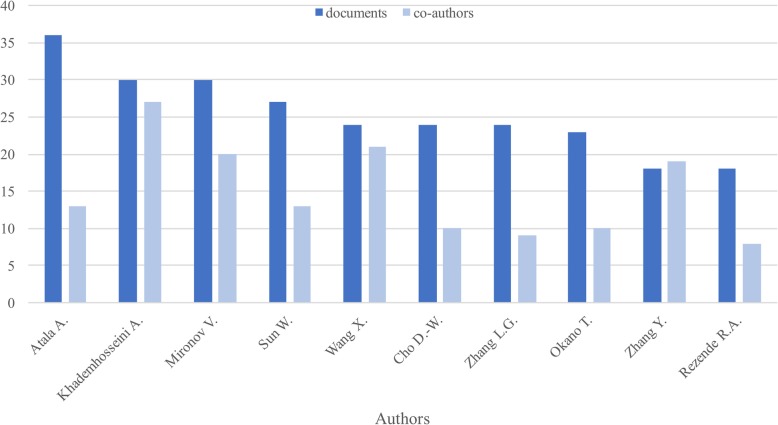
Number of documents and co-authors of the top ten authors in bioprinting

From the analysis, only ten authors were found to have more than 18 documents, as seen in Fig. 2, where the number of documents and the number of co-authors for each of them are shown. The author with more documents falling in the threshold defined is Atala A. with 36 documents and 13 co-authors. The following author, Khademhosseini A., had a total of 30 documents and more than double of collaborations for the first author, 27 co-authors, being the one with more connections. Mironov V. was in third place with 30 documents, and 20 co-authors. A Pearson correlation analysis was performed to determine the relationship between documents and co-authors, and a weak positive correlation was observed, as the Pearson correlation coefficient had a value *r* = 0.29 for the top ten authors, stating a lack of relationship between the number of co-authors and the number of publications.

**Fig. 3:**
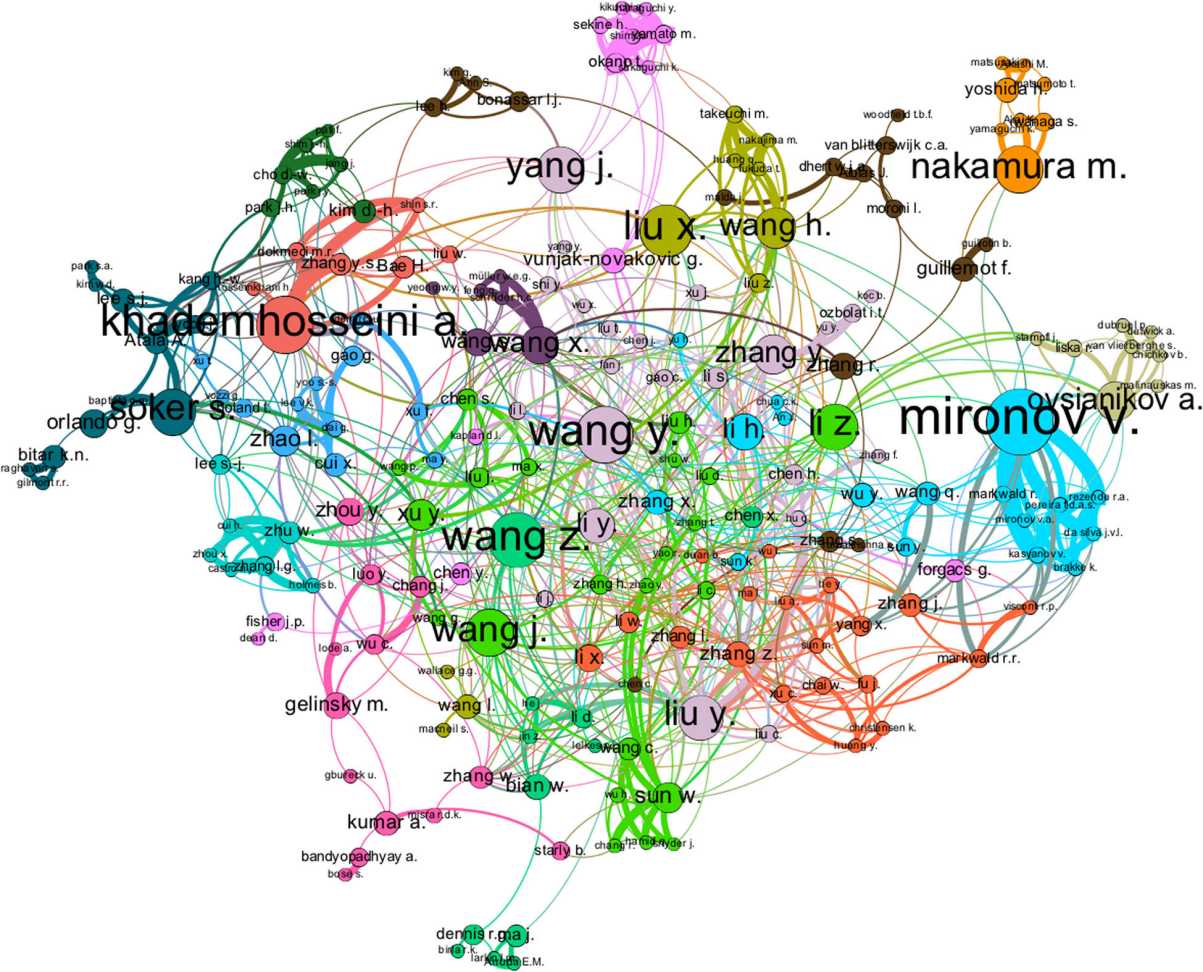
Co-authors network map, the authors names were normalized with lower case letters

Figure 3 shows the network map of the author’s collaboration, where the nodes’ size is proportional to their BC value. The nodes representing the authors were grouped in a total of 17 clusters. From the BC calculation, the most prolific author, Atala A., was at the Wake Forest Institute for Regenerative Medicine from the Wake Forest University School of Medicine, Winston Salem, United States when the information was gathered (15 November 2017). According to Scopus altmetrics, this author had an h-index of 89, 850 documents published, and a total of 17,376 citations working with 150 co-authors at the time of the analysis (see Table 2). On the other hand, under the inclusion terms, this author published a total of 36 documents on the topic analysed, having 13 connections, 2851 citations, and a betweenness centrality value of 370.9.

The second most prolific author found is Khademhosseini Ali L.I., affiliated with the Brigham and Women’s Hospital, Department of Medicine, Boston, United States, when the data was collected. This author had an h-index of 88, a total of 645 papers, with a total of 16,704 citations and 150 co-authors, as stated in the Scopus altmetrics. Considering the inclusion terms, this author accounted for 30 documents, 27 connections, 3047 citations, and a betweenness centrality of 2104.9 (see Table 2).

The third author was Mironov V., from the Laboratory for Biotechnological Research ‘3D bioprinting solutions’, Moscow, Russian federation. The Scopus altmetrics showed that this author had 105 papers, 3231 cites, and an h-index of 31, co-authoring with 150 people. In this analysis, the author accounted for 30 documents, 20 links, 1009 citations, and a betweenness centrality of 2754.9 (see Table 2).

According to the network map and the BC calculations, Mironov V. was stated as the author with a higher influence in the knowledge flow of the collaboration network, as it had the higher BC, followed by Khademhosseini A. While Mironov was affiliated to a biotechnological research laboratory, Atala and Khademhosseini were associated to two of the top ten research departments in bioprinting found on this analysis.

The authors ranked by the experts were compared with the most influential authors disclosed in this study, as it can be seen from Table 3.

Three of the ten top authors in this scientometric study were considered as influential by the experts consulted, Atala A., Mironov V., and Wei Sun; who were listed among the top five authors in both cases. The top three authors from this study, who are listed in Table 3, are also the main influential authors with a higher BC (see Table 2).

**Table 2:** Comparison of the Scientometric information between Scopus and the analysis performed to the top three authors with 5 or more documents with 10 or more citations

Author	Documents		Connections		Citations		BC	h-index
	Scopus	Threshold	Scopus	Threshold	Scopus	Threshold		
Atala A.	850	36	150	13	17,376	2851	370.9	89
Khademhosseini Ali L.I.	645	30	150	27	16,704	3047	2104.9	88
Mironov V	105	30	150	20	3231	1009	2754.9	31

**Fig. 4:**
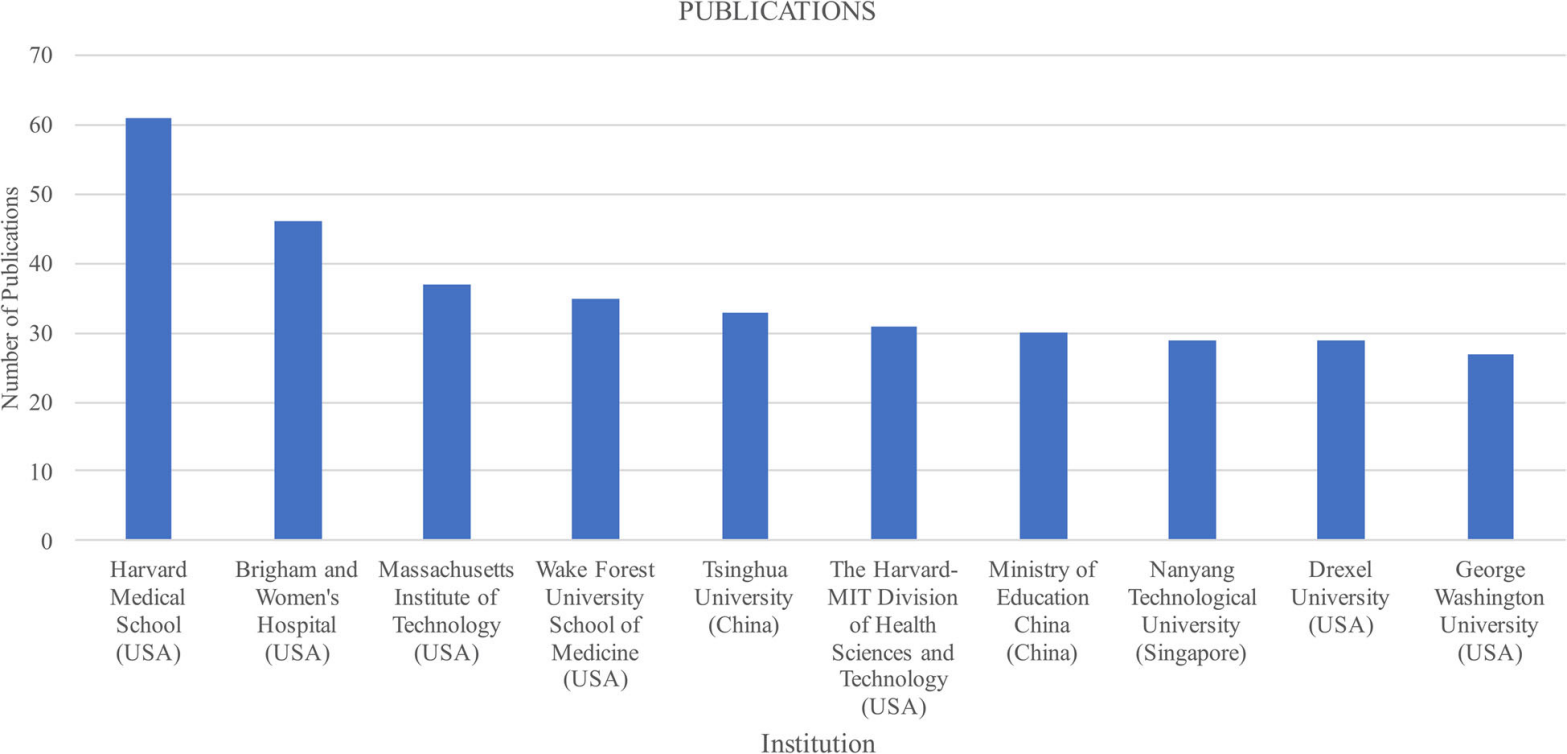
Top ten institution and number of publications in bioprinting

Institutions’ research efforts can be better estimated by the number and the quality of their publications, therefore the affiliations with more publications on bioprinting are here analysed. A total of 1760 affiliations were identified in the information obtained for the 2088 documents, with a median of 10 publications per institution and a standard deviation of 7.8. The top ten affiliations with more publications in bioprinting are presented in Fig. 4. An interesting fact is that seven of the top ten are based in the United States, two of them are in China and one in Singapore.

Remarkably, four of the 7 affiliations in the United States are located in Massachusetts, and three of them have a higher number of publications within the threshold. The Harvard Medical School is the institution with more publications in the analysis here presented, with 61 documents, followed by the Brigham and Women’s Hospital, with 46 documents. Both affiliations are located at the Longwood Medical and Academic Area, a medical campus in Boston with a strong life science cluster [56]. The Brigham and Women’s Hospital, is an institution joint to the Harvard Medical School and holds the second largest hospital-based program in the world, pioneering in the heart valve operation and the world’s first solid organ transplant [57].

The Massachusetts Institute of Technology (MIT), located in Cambridge, MA, was found to be in third place on papers related to the bioprinting, presenting 37 documents within the threshold defined. This institute holds the fifth place in the World University Ranking 2016–2017 of the Times Higher Education [58]. The fourth institution found with more publications is the Wake Forest University School of Medicine, an academic medical centre ranked among the best in the United States, promoting research in medical areas [59]. This affiliation shows 35 documents. Tsinghua University is placed in fifth place, with 33 publications. This institution was on the 35th place on the Times Higher Education World University Rankings 2017 [58].

**Fig. 5:**
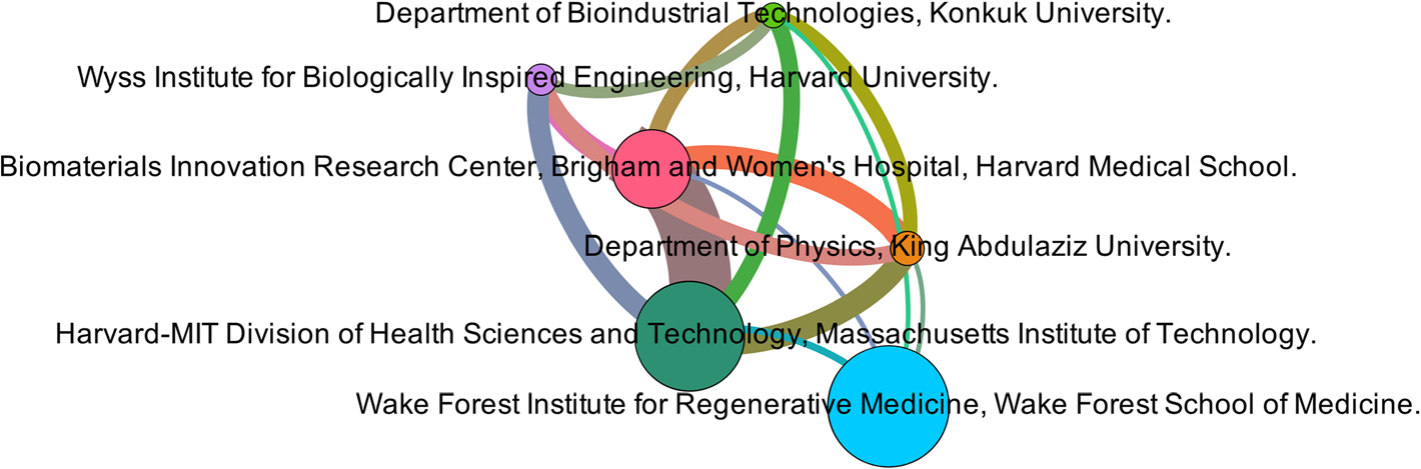
Network map of the collaboration between affiliations

For the affiliations’ analysis, a total of 20 out of the 1760 institutions met the inclusion requirements, which at least five documents with at least five citations each. However, only six affiliations within this threshold were found to have a collaboration with external institutions. Figure 5 depicts the collaboration network between these institutions, the size of the nodes is proportional to their number of documents, while the thickness of the connection line is proportional to the strength of the link, which is equal to the number of documents they have co-authored. Within the inclusion limits above stated, the Harvard-MIT Division of Health Science has the first position having 37 papers, followed by the Wake Forest Institute for Regenerative Medicine with 28 documents and the Biomaterials Innovation Research Centre from the Brigham and Women’s Hospital with 26 documents.

On the other hand, the network map shows four institutions with 5 collaborations. These institutions are the Biomaterials Innovation Research Centre, at the Brigham and Women’s Hospital from the Harvard Medical School, United States, the Harvard-MIT Division of Health Sciences and Technology at the Massachusetts Institute of Technology, United States, the Department of Physics at the King Abdulaziz University, Saudi Arabia, and the Department of Bioindustrial Technologies, Konkuk University, South Korea. The remainder institutions have four connections, the Wyss Institute for Biologically Inspired Engineering, Harvard University, United States and the Wake Forest Institute for Regenerative Medicine, Wake Forest School of Medicine. Interestingly, three of the six affiliations are located in Massachusetts and two of them are associated to the University of Harvard, the second-best research university from the United States [60]. This institution has a close collaboration with the top institute in the United States, the Massachusetts Institute of Technology [58]. Both institutions have founded the Harvard-MIT Division of Health Sciences and Technology, associated to the Massachusetts Institute of Technology. This institute is the one with more citations, 1454, which also has a strong collaboration with the Biomaterials Innovation Research Centre, which has 1099 citations. These affiliations are followed by the Wake Forest School of Medicine with 858 citations.

The low number of institutions and the high degree of connectivity among them is reflected on the computation of the BC for each institution. The four affiliations with five links each had the same BC centrality, 0.25, calculated with Gephi’s statistic tools; this low value means that all the affiliations share the same importance for the network. On the other hand, the remaining two affiliations have four connections, and a BC equal to zero, not contributing significantly to the network.

## Discussion

The identification leading authors and affiliations and their collaborations were the main factors to be determined in this study. The use of the text mining software developed was found to have a significant contribution to the identification of keywords to design the search queries. This step was crucial to include the complete set of publications discussing the topic of bioprinting. The search queries created included a wide range of terms and synonyms that are used in articles on topics related to bioprinting. An exponential growth of publications per year was observed from the data of the documents obtained. Furthermore, as one of the main goals of this study was to identify the most influential authors working in bioprinting, the analysis, including author disambiguation, was exclusively based on the publications’ information specifically obtained from the Scopus database. Although there are more scientific databases available, Scopus was selected because it also contains non-English coverage and its altmetrics tools were used for a quick overview of the results. Although Databases such as Web of Science cover records back to 1900, we analysed only a specific period, which could be covered by Scopus. Although results provide the appraisal of one of the most complete scientific databases available, Scopus, the information obtained can be further enhanced by analysing data obtained from more databases to support the findings. The author disambiguation was performed manually for the authors with good agreement; however, this procedure can be improved by including tools such as similarity of pairs or features contribution. In this analysis it was observed that although a high number of authors were engaged in the advancement of the scientific output on bioprinting, only a small percentage have a remarkable productivity on the topic. Furthermore, there was found a slight association between the number of documents and the number of collaborations. Co-author analysis has contributed to the identification of the intellectual structure of fields and specialties [2] and to identify research groups [3]. In this research, the network map analysis was enhanced with the calculation of BC to identify the authors with more publications and the most influential authors and institutions working in bioprinting. Although some of the authors might be regarded as scientists with a higher rank or seniority, this classification was beyond the scope of this study.

The number of affiliations working in bioprinting was found to be high, as expected. However, only a small portion of them fulfil the inclusion requirements for the analysis. The most prolific institutions that came across in this study, such as Nanyang Technological University, MIT, and Tsinghua University, were also previously reported to be among the three most prolific in [15]. Moreover, a reduced number of collaborations between the institutions in the threshold was found, an unanticipated outcome for a multidisciplinary technology. The institutions with more publications, The Harvard Medical School and the Brigham and Women’s hospital, were two of the top ten Universities in the World University Ranking [58], which have established a research centre close to both institutions. Besides, the strategic geographical position of the affiliations to promote collaborations has been observed as important to encourage scientific production. But not only the institutions located closely, namely the Harvard Medical School, Brigham and Women’s Hospital and the Division of Health Science, were found to have strong collaborations, also the Department of Bioindustrial Technologies of the Konkuk University and the Department of Physics, of the King Abdulaziz University exhibited a high degree of collaboration. This illustrates that geographical positioning is an important factor to collaborate, but it is not crucial.

The method here presented involved the overall static analysis of collaborations over a ten-year period, as the change in time for both kinds of collaboration, those among authors and those among affiliations, were not analysed for different periods. Furthermore, the results here shown concern the general approach of bioprinting domain, where a wide range of methodologies and technologies are involved without special emphasis on any particular methodology. In this sense, any agreement on the most influential authors and institutions is more difficult to reach. The threshold set in this study was used to determine the most influential authors and institutions in bioprinting, taking into account also the departments of affiliation, thus differing from the analyse made by Rodriguez-Salvador et al. [7], where only countries and institutions were considered. Regarding affiliations, insights obtained in the analysis are consistent with the institutions reported in by Rodriguez-Salvador et al. [7]. Both analyses, this analysis and the one reported by Rodriguez-Salvador et al. differ from the list provided by the experts, shown in Table 3. The threshold was defined to include only those authors and affiliations with cites above the average, and this influenced the network map analysis, by reducing significantly the number of nodes in the map. Another aspect that influenced the results was the exclusion of those nodes with no connections. These nodes were neglected in this study as zero links on the maps mean zero BC and do not have any effect in the overall results. Furthermore, another possible reason for the difference between the list provided by experts and the results here disclosed, is that expert trajectories can have a subjective influence to define the most influential authors in bioprinting.

**Table 3:** Comparison of authors in bioprinting ranked by experts versus the most influential authors disclosed in this study

Rank	List of most influential authors provided by experts		List of most influential authors found on this study	
	Author	Institution	Author	Institution
1	Atala A.	Wake Forest Institute for Regenerative Medicine	Atala A.	Wake Forest Institute for Regenerative Medicine
2	Mironov V.	Laboratory for Biotechnological research	Khademhosseini Ali L.I.	Brigham and Women’s hospital
3	Malda J.	Utrech University	Mironov V.	Laboratory for Biotechnological research
4	Derby B.	University of Manchester	Sun W.	Drexel University and Tsinghua University
5	Sun W.	Drexel University and Tsinghua University	Wang X.	Tsinghua University
6	Lewis J.	Harvard	Cho D. W.	Pohang University of Science and Technology
7	Yoo J.	Wake Forest Institute for Regenerative Medicine.	Zhang L. G.	George Washington University
8	Woodfield T.	University of Otago	Okano T.	Tokyo Women’s Medical University
9	Dalton P.	University of Wurzburg	Zhang Y.	Brigham and Women’s hospital
10	M. Zanobi-Wong	ETH Zurich	Rezende R. A.	Centre for information Technology Renato Archer

In this research, we also disclosed the six main institutions working on bioprinting and their collaboration network. The use of network analysis and the calculation of the BC was decisive to find the authors with a higher degree of influence on the topic. However, the identification of research areas of both authors and affiliations was out of the scope of this research, and this can be investigated when looking for R&D opportunities for innovation in bioprinting. This research set the basis to determine collaborations and their position in a scientific network. The knowledge obtained in our research can provide support to researchers and stakeholders looking for engagement in R&D projects on bioprinting.

## Conclusions

Network map analysis was used here to identify the most prominent institutions and authors. A threshold was defined to disclose authors and organisations with a higher network of collaboration, identifying authors with more publications. Moreover, the Betweenness Centrality calculation allowed us to identify the most influential authors and institutions working on bioprinting. The outcomes obtained can give strength to the perception of the collaborations in bioprinting technologies. Although the global research community in bioprinting has grown, the most influential affiliations and authors are located in the United States. The top three authors have more than 29 articles each within the threshold established. From the authors’ network map analysis, it was observed that there is no direct correlation between the BC, number of documents, and connections, as the one with more documents in the threshold was the one with less connections and the lower BC value. The affiliations with more publications are members of the top universities in the United States and are part of medical research programs. Individuals interested in the development of bioprinting can benefit from the information here disclosed to perform a trend analysis on the institutions hereby mentioned. And identifying core technologies that have led them to success. The findings of this study can offer valuable information to be used in systematic approaches to support the decision making of researchers and stakeholders.

## Data Availability

The datasets generated and/or analysed during the current study are available in the Open Science Framework repository, https://osf.io/ez7mv/ . Named bioprinting_scopus_data_10.csv,

## Appendix

### Search query

TITLE-ABS-KEY (((((3d OR 3-d OR three-dimensional) W/1 (bioprint* OR engineer* OR print* OR tech* OR fabricat* OR process* OR manufact* OR building OR built)) OR ((bio-engineer* OR bioengineer* OR biofabricat* OR bioprint* OR biotech* OR biomanufact*)) OR (bioadditive W/1 manufact*) OR (3d AND bioprint*))) W/5 (scaffold* OR construct* OR spheroid* OR channel* OR structure* OR matr* OR crosslinking OR block* OR aggregate* OR sheet* OR biomim* OR bioactiv* OR biohybrid* OR bioresorbable OR bioscaffolds OR biosensors OR bioassembl* OR bioartificial OR bioerodible OR biopatterning OR biopaper OR microextrusion) W/5 (cell* OR tissue* OR stem OR multicellular OR organ* OR biolog* OR embryonic OR vascular OR vessels OR colagen OR bone* OR osseo* OR adipose OR vascular OR cardiac OR heart OR cartilage* OR muscle) AND NOT (“data storage” OR photonic OR pcl OR “social capital”)) AND (PUBYEAR > 2000 AND PUBYEAR < 2018).

## Abbreviations

BC: Betweenness Centrality
CI: Competitive Intelligence
CSV: Comm Separated Values
CTI: Competitive Intelligence
DL: Digital Libraries
FICM: Field Independent Cluster Model
MIT: Massachusetts Institute of Technology
R&D: Research and Development
VOS: Visualisation of singularities
